# Effect of metachronous primary and secondary solid cancers in patients with multiple myeloma: a retrospective study from a single-center

**DOI:** 10.3389/fimmu.2025.1516471

**Published:** 2025-03-10

**Authors:** Yunfei Ji, Hujun Li, Huanxin Zhang, Hai Cheng, Ying Wang, Kailin Xu, Zhenyu Li

**Affiliations:** ^1^ Department of Hematology, The Affiliated Hospital of Xuzhou Medical University, Xuzhou, Jiangsu, China; ^2^ Blood Diseases Institute, Xuzhou Medical University, Xuzhou, Jiangsu, China; ^3^ Key Laboratory of Bone Marrow Stem Cell, Xuzhou Medical University, Xuzhou, Jiangsu, China

**Keywords:** multiple myeloma, solid cancer, survival, risk, immunomodulator

## Abstract

**Statement of translational relevance:**

Effects of metachronous primary malignant solid tumor (MPMST) on survival risk and prognosis of multiple myeloma (MM) and differences between MPMST occurring before and after MM remains unclear. Use of well-characterized clinical information of individual patient, we found that older patients with MM (≥ 65 years) had a higher risk of developing MPMST. Patients with MM and MPMST including male patients, aged ≥ 65 years and those with ISS stage III had a worse prognosis. The top three solid cancers occurred before and after MM were the lung, thyroid, and breast cancer. These findings provide detailed information for the precise treatment of patients with MM and MPMST.

**Objective:**

To analyze the effects of MPMST on MM and the risk difference of MPMSTs occurring before and after MM.

**Methods:**

Retrospective data from patients with MM and MPMST, including sex, age, immunoglobulin isotype, ISS stage, and therapy, were collected from 2015 to 2023. Differences in variables, risk, and survival were compared using the *χ²* test, logistic regression analysis and the Cox model, respectively.

**Results:**

The 34 (1.57%) patients with MM and MPMST identified from a total of 2167 MM patients had a shorter overall survival. The survival risk was higher in male patients with MM and MPMST (HR: 3.96, 95% CI: 1.05 -14.96), in those aged ≥ 65 years (HR: 3.30, 95% CI: 1.41 -7.71), and with ISS stage III (HR: 4.08, 95% CI: 0.81-20.65). Patients with MM subsequent to CAR-T cell therapy had neither enhanced incidence rates of second solid cancers nor had longer overall survival time. Furthermore, the top three solid cancers occurred before or after MM were lung, thyroid, and breast cancer.

**Conclusion:**

Male patients, aged ≥ 65 years and MM patients with ISS stage III and MPMST had a worse prognosis.

## Introduction

Growing evidence indicates that prolonged survival of in patients with multiple myeloma (MM) patients can be attributed to the introduction of agents such as proteasome inhibitors(PIs), immunomodulatory drugs(IMiDs), monoclonal antibodies, autologous stem-cell transplantation (ASCT) and chimeric antigen receptor T-cell (CART- cell) therapy (1–5) ; however, treatment-related secondary hematological diseases caused by lenalidomide, bortezomib, high-dose chemotherapy, ASCT, and CAR T-cell therapy in patients with MM have attracted increasing attention (6–10). The risk of secondary solid cancer in MM patients with longer lifespan has been described (11–21). However, less is known about the risk and prognosis of patients with MM and metachronous previous or secondary primary solid tumor, namely MPMST.

Multiple primary malignant tumor including MPMST, refer as two or more histologically validated primary malignant tumors that occur in a patient, which may be derived from the same or different organs and different systems, excluding the metastasis of initial primary cancers (19, 22–26). An interval between two primary malignancies of more than two months is commonly defined as metachronous multiple primary tumors according to the recommendation of the Surveillance, Epidemiology and End Results (SEER) Program; Multiple primary malignant tumor occurring within two months are considered synchronous tumors (23, 27).

The incidence rate of MPMST varies in different countries, ranging from 0.52% to 11.7% (28) and most MPMSTs are double primary MPMST. An increased risk and worse prognosis were found in patients with MM and secondary hematological or secondary solid tumors (11–21). Jonsdottir et al. demonstrated that a prior cancer diagnosis is a risk factor for the development of subsequent cancers in multiple myeloma patients (24). Similarly, several long-term population-based clinical trials have demonstrated that patients with MM and additional primary malignancies, especially older aged individuals and males, had a worse prognosis (18, 19). These findings suggest an impact of multiple primary malignant tumors in patients with MM. However, the effects of MPMST diagnosed before and after MM on the survival risk of MM have not been fully investigated.

Here, using well-characterized individual clinical information, we identified thirty-four patients with MM and MPMST with histopathological validation from 2167 MM patients, including 11 (32.35%) patients with MPMST diagnosed after MM, and 23 (67.65%) patients with MPMST diagnosed before MM. The rate of occurrence of MPMSTs according to sex, age, ISS stage, and treatment regimens including IMiDs, PIs, chemotherapy, ASCT, and CAR -T cell therapy was evaluated in patients with MM. Differences in survival were compared between MM patients with or without MPMSTs, differences in morbidity between MPMSTs diagnosed before and after MM, as well as morbidity and mortality of primary solid tumor occurring before and after the diagnosis of MM, were also evaluated, which provides detailed information for the precise treatment of patients with MM and MPMST.

## Patients and methods

### Patients and grouping

We conducted a retrospective study by collecting histologically confirmed MM patients at the Affiliated Hospital of Xuzhou Medical University from July 1, 2015 to December 31, 2023 (ChiCTR2100048888). This work was approved by the Ethics Committee of the Affiliated Hospital of Xuzhou Medical University. Written informed consents were available prior to enrollment in the study in accordance with the Declaration of Helsinki.

MM was diagnosed according to International Myeloma Working Group (IWMG) criteria (29). Clinical staging of MM was based on Durie & Salmon (DS) or the International Staging System (ISS) (12, 30). Solid tumor diagnosis and staging were based on histological examination and TNM staging. Sixty-six patients with MM without MPMSTs were selected as the controls after matching for age, sex and year of diagnosis to patients with MM and MPMSTs according to previous studies (19, 22). Subjects with additional hematological and additional primary solid tumors diagnosed within two months, or with metastatic malignancy are excluded (23, 27). Clinical information mainly included age, sex, Immunoglobulin Isotype, ISS stage, levels of β_2_-MG and lactate dehydrogenase (LDH) at study entry, therapies for MM and solid tumor, and the time free from the second tumor to the first tumor (TF2T) were evaluated (31).

### Treatment

MM-related treatment mainly consisted of CART- cell therapy, ASCT, PIs and/or IMiDs combined with dexamethasone, such as bortezomib and dexamethasone (Vd), lenalidomide and dexamethasone (Rd), lenalidomide, bortezomib and dexamethasone (RVd), bortezomib, cyclophosphamide, dexamethasone (VCd), bortezomib, thalidomide and dexamethasone (VTd), thalidomide, cyclophosphamide and dexamethasone (TCd), or anthracycline-based induction (idarubicin/dexamethasone or VAD), or daletumab monoclonal antibody-based regimen. CART-cell therapy can be selected (29, 32). Solid tumor-related treatment consists of surgical operation, chemotherapy or combined treatment.

### Outcomes

For MM patients with or without MPMST, the overall survival time was estimated from the date of the first diagnosis of MM or additional cancer until death or the end of the study (31 December 2023), whichever occurred first. For patients with MM and additional solid cancers, TF2T was calculated to compare differences in occurrence between MPMSTs before and after MM. The diagnosis of first and second cancer was validated by histological examination and/or computed tomography (CT).

### Follow-up

Patients with histological diagnosis of MM and additional primary solid tumor received regular follow-up, which included hospital records of inpatients and a telephone follow-up for outpatients (12, 32). The follow-up deadline of these patients treated at our institution was at 31 December, 2023 or at the time of Death (for any reason).

### Statistical analysis

Statistical analysis was performed using SPSS (v.22.0, SPSS Inc. Chicago, IL, USA) and GraphPad Prism v.8.0 (GraphPad Software, La Jolla, CA). Pearson’s chi-square test or Fisher exact tests were used to compare the differences among categorical variables in different groups. The risk factors related to MPMST were evaluated by unconditional logistic regression analysis and odds ratio (OR) along with 95% confidence intervals (CI). The Kaplan-Meier method and logarithmic rank test were employed to compare differences in survival time. Cox proportional hazards model was adopted for the univariate and multivariate analysis of OS. *P* < 0.05 was considered statistically significant.

## Results

### Characteristics in MM patients with or without MPMSTs

**Figure 1:**
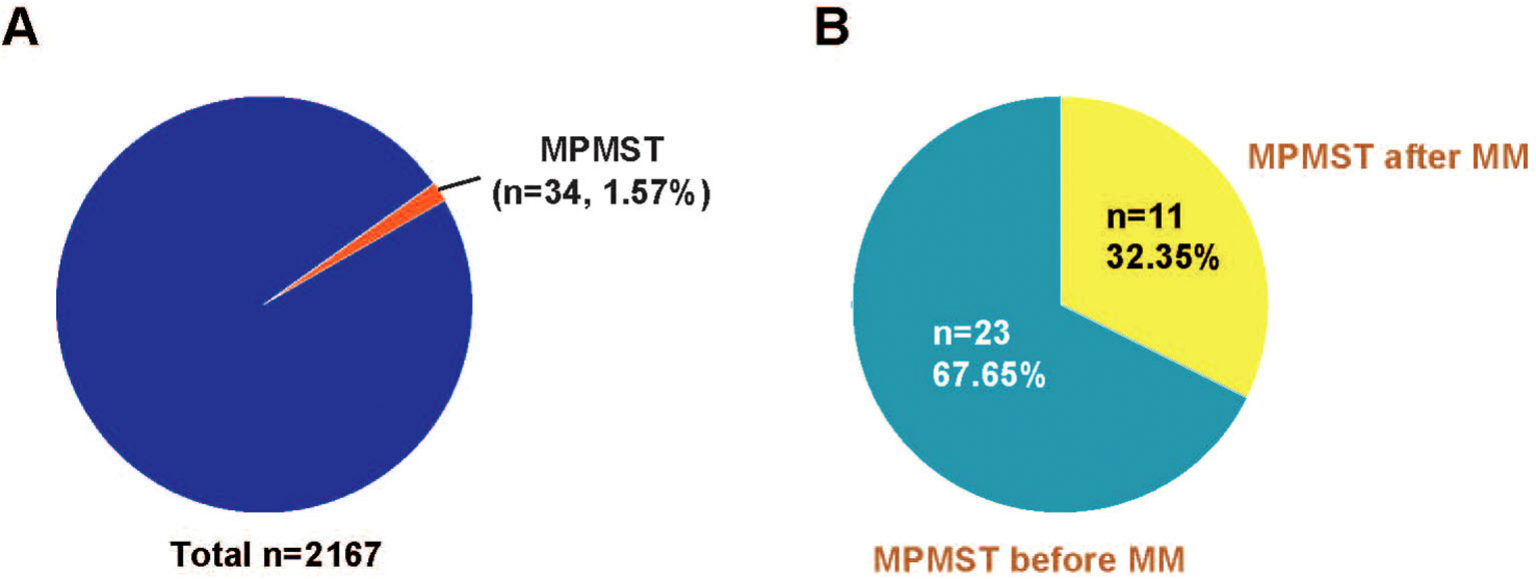
The incidence rate of MPMST in MM and the rates of MPMSTs occurred before and after MM. **(A)** The incidence rate of MPMSTs in MM. **(B)** The rates of MPMSTs occurred before and after MM. MPMST, metachronous primary multiply solid tumor; MM, multiple myeloma.

As illustrated in **Figure 1**, among 2167 patients with MM enrolled, 34 patients with MPMST were identified, the incidence rate of MPMST was 1.57%. Of which, MPMST diagnosed after MM and before MM were 32.35% and 67.65%, respectively (**Figure 1**). The median age of MM with MPMSTs was 67.0 years. Patients with MM and MPMST were more likely to have higher β_2_-MG levels than those without MPMSTs (28.8% vs. 50.0%, *P* = 0.04, **Table 1**). The rate of TP53 mutation in patients with MPMST was 5.9% (2/34). There was no significant difference between MM patients with or without MPMSTs in terms of the number of monoclonal globulin types, stage of ISS, survival rate and LDH levels collected at the time of study entry, or maintenance therapy for MM (**Table 1**).

**Table 1:** Differences in the distribution across characteristics in MM patients with or without MPMST.

Characteristics	MM	with MPMST				
	(n=66)	Total (n=34)	*P*	before MM (n=23)	after MM (n=11)	*P*
**Gender, *n* (%)**			0.33			0.98
Male	34 (51.5)	20 (58.8)		13 (56.5)	7 (63.6)	
Female	32 (48.5)	14 (41.2)		10 (43.5)	4 (36.4)	
**Age (years), *n* (%)**			0.07			**0.04**
< 65	36 (54.5)	12 (35.3)		5 (21.7)	7 (63.6)	
≥ 65	30 (45.5)	22 (64.7)		18 (78.3)	4 (36.4)	
**Immunoglobulin Isotype, *n* (%)**			0.23			0.25
IgG	25 (37.9)	17 (50.0)		12 (52.2)	5 (45.4)	
IgA	12 (18.2)	9 (26.5)		4 (17.4)	5 (45.4)	
IgD	4 (6.1)	1 (2.9)		1 (4.3)	0 (0.0)	
Light chain	21 (31.8)	7 (20.6)		6 (26.1)	1 (9.1)	
No secretory	4 (6.1)	0 (0.0)		0 (0.0)	0 (0.0)	
**ISS stage at study entry, *n* (%)**			0.20			0.51
I	18 (27.3)	15 (44.1)		10 (43.5)	5 (45.4)	
II	21 (31.8)	10 (29.4)		8 (34.8)	2 (18.2)	
III	27 (40.9)	9 (26.5)		5 (21.7)	4 (36.4)	
**β_2_-MG, *n* (%)**			**0.04**			0.71
Normal	47 (71.2)	17 (50.0)		12 (52.2)	5 (45.4)	
High	19 (28.8)	17 (50.0)		11 (47.8)	6 (54.6)	
**LDH, *n* (%)**			0.35			0.59
Normal	45 (68.2)	21 (61.8)		13 (56.5)	8 (72.7)	
High	21 (31.8)	13 (38.2)		10 (43.5)	3 (27.3)	
**TF2T (months), *n* (%)**						**0.04**
< 36	–	–		8 (34.8)	8 (72.7)	
≥ 36	–	–		15 (65.2)	3 (27.3)	
Therapy for MM						
Immunomodulator	61 (92.4)	24 (70.6)		–	7 (63.6)	
Protease inhibitor	64 (97.0)	27 (79.4)		–	10 (90.9)	
Chemotherapy	58 (87.9)	26 (76.5)		–	9 (81.8)	
ASCT	16 (24.2)	8 (23.5)		–	3 (27.2)	
CAR-T cell therapy	10 (15.1)	6 (17.6)		–	2 (18.2)	

MM, multiple myeloma; MPMST, metachronous primary malignant solid tumor; LDH, lactate dehydrogenase; β_2_-MG, β_2_ macroglobulin; TF2T, time free to second tumor; Chimeric antigen receptor T ASCT, autologous stem cell transplantation; CAR-T cell therapy, Chimeric antigen receptor T cell therapy. The bold values mean a significant difference in two groups.

**Figure 2:**
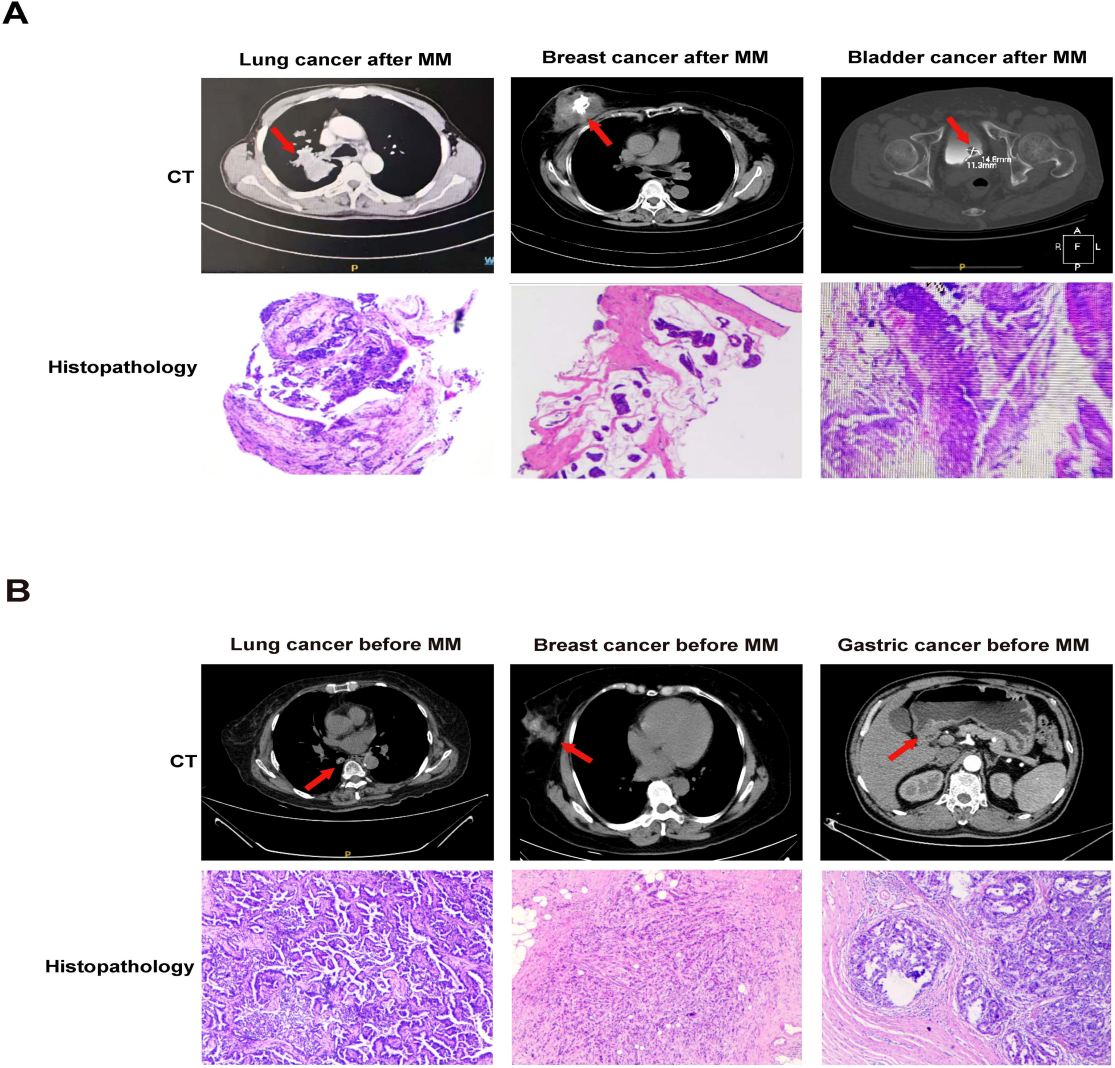
CT and histopathology in MPMSTs occurred after and before MM. **(A)** CT and histopathology of second lung cancer, breast cancer, bladder cancer in MM patients. **(B)** CT and histopathology of prior lung cancer, breast cancer, gastric cancer in MM patients.

Plasmacytoma, particularly EMP at relapse can be found in the liver, kidney, lymph nodes, breast, and maybe misdiagnosed as primary solid tumors (33, 34). Our CT and histopathology results documented that solid cancer occurring before or after MM mainly consisted of lung, breast, bladder, prostate and gastric cancers (**Figure 2**).

### Risk factors associated with the appearance of MPMST in patients with MM

A population-based study revealed that combined treatment may increase the risk of secondary cancers in patients with MM (11). However, the data from Munker et al. indicated that most additional cancers diagnosed that occurred in patients with MM were not associated with MM treatment. The mechanisms underlying MPMST in MM are complex and involve immunologic, genetic, or environmental factors (35). Our results indicated that MM patients aged ≥ 65 years or with higher β_2_-MG levels had an increased risk of MPMSTs (age: OR: 7.47, 95% CI: 2.95-18.92. β_2_-MG: OR: 3.21, 95% CI: 1.18-8.68. **Table 2**).

**Table 2:** Risk factors related to the occurrence of MPMST in MM patients.

Characteristics	MM (n=66)	with MPMST (n=34)	MPMST after MM (n =11)	MPMST			MPMST after MM		
				OR	95%CI	*P*	OR	95%CI	*P*
**Gender**				0.66	0.29-1.52	0.33	1.86	0.50**-**6.96	0.36
Male	34(51.5)	20(58.8)	7(63.6)						
Female	32(48.5)	14(41.2)	4(36.4)						
**Age(years)**				7.47	2.95-18.92	**0.00**	1.46	0.39-5.46	0.57
<65	36(54.5)	12(35.3)	7(63.6)						
≥65	30(45.5)	22(64.7)	4(36.4)						
**ISS stage**				0.47	0.20-1.13	0.09	0.72	0. 33-1.56	0.40
I	18(27.3)	15(44.1)	5(45.4)						
II	21(31.8)	10(29.4)	2(18.2)						
III	27(40.9)	9(26.5)	4(36.4)						
**β_2_-MG**				3.21	1.18-8.68	**0.02**	0.34	0.09-0.24	0.10
Normal	47(71.2)	17(50.0)	5(45.4)						
High	19(28.8)	17(50.0)	6(54.6)						
MM therapy									
Immunomodulator	61(92.4)	24(70.6)	7(63.6)	–	–	–	6.97	1.51-32.19	**0.01**
Protease inhibitor	64(97.0)	27(79.4)	10(90.9)	–	–	–	3.20	0.26-38.64	0.36
Chemotherapy	58(87.9)	26(76.5)	9(81.8)	–	–	–	1.61	0.29-8.83	0.58
ASCT	16(24.2)	8(23.5)	3(27.2)	–	–	–	0.85	0.20-3.61	0.83
CAR-T cell therapy	10(15.1)	6(17.6)	2(18.2)	–	–	–	0.83	0.27-2.56	0.75

MM, multiple myeloma; MPMST, metachronous primary malignant solid tumor; β2-MG, β2 macroglobulin; ASCT, autologous stem cell transplantation; CAR-T cell therapy, Chimeric antigen receptor T cell therapy. The bold values mean a significant difference in two groups.

Furthermore, patients with MM receiving immunomodulators were associated with an increased risk of developing a second solid tumor (OR: 6.97, 95%CI: 1.51 -32.19) (**Table 2**). No significant differences in the risk of second solid tumor in patients with MM treated with PIs, chemotherapy, ASCT, and CAR-T cell therapy were observed (**Table 2**).

### Risk factors related to the survival in patients with MM and MPMST

**Figure 3:**
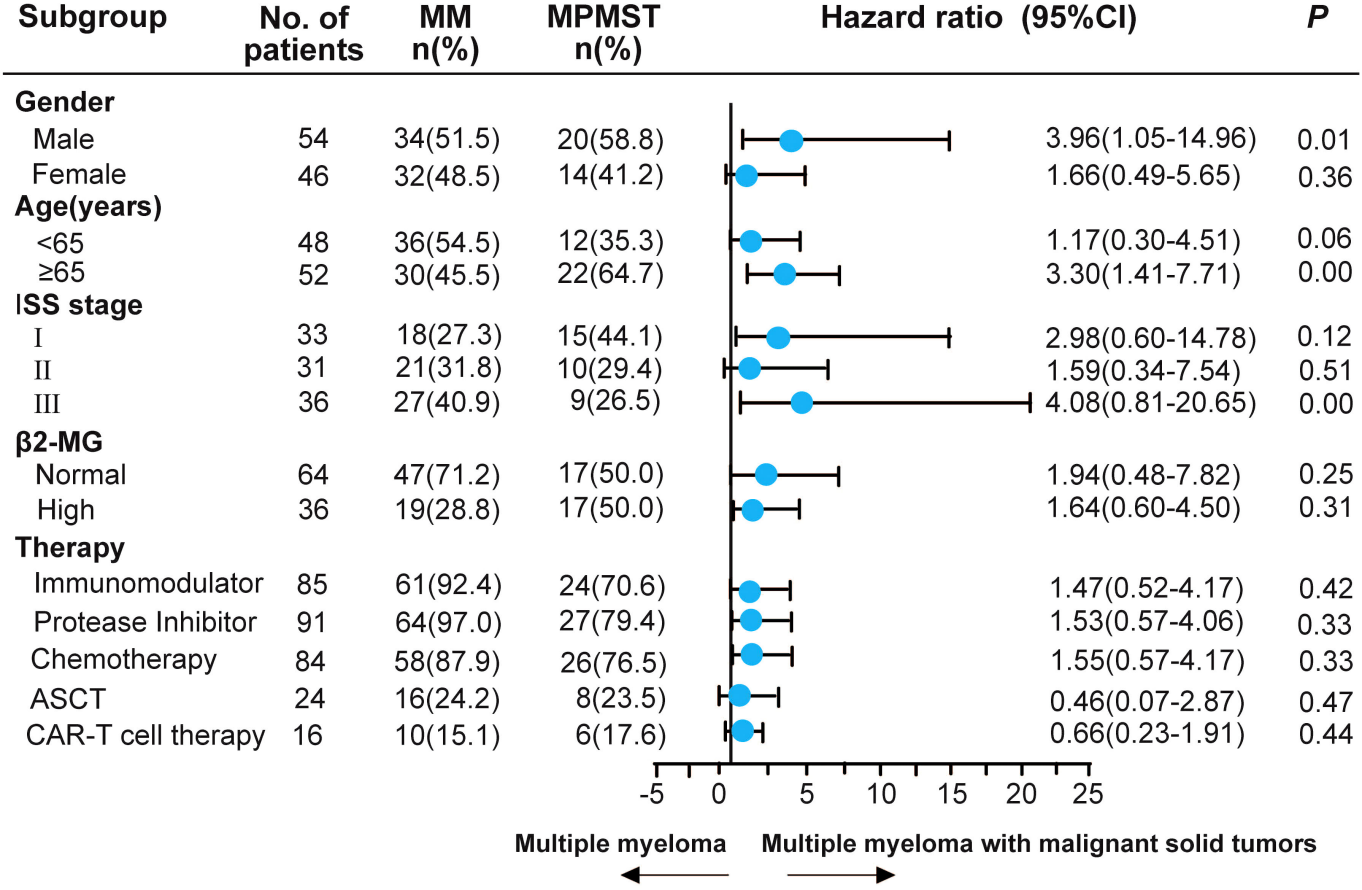
The subgroup analysis of risk factors associated with the survival of MPMST. Blue dots represent the observed Hazard ratios, and the lines extending from the dots are the 95% CI for these proportions. 95% CI: 95% confidence interval. ASCT, autologous stem-cell transplantation; β_2_-MG, β_2_ macroglobulin.

The published literature revealed that previous malignancies, including solid cancer, negatively affect survival in patients with MM (24). Risk factors related to survival were investigated in patients with MM and MPMST. A significantly higher survival risk was found in male patients with MPMST (HR: 3.96, 95% CI: 1.05 -14.96), patients aged ≥ 65 years (HR: 3.30, 95%CI: 1.41 -7.71), and in those with stage III ISS (HR: 4.08, 95% CI: 0.81-20.65). No significant differences were identified in survival risk related to treatment including the use of IMiDs, PIs, ASCT, and CAR-T cell therapy between MM patients with and without MPMST (**Figure 3**).

**Figure 4:**
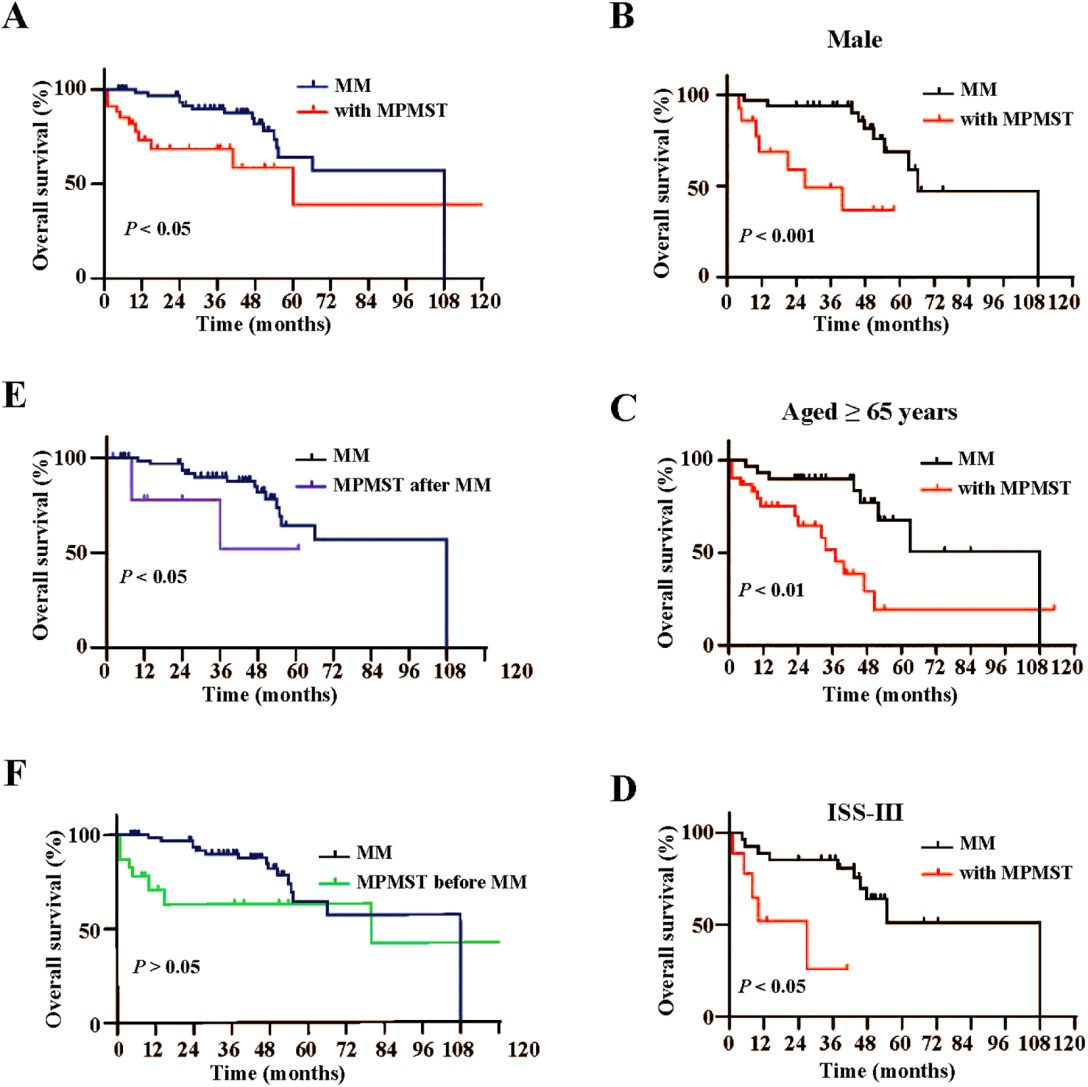
Kaplan-Meier analysis of overall survival in subgroups between MM patients with and without MPMST. **(A)** Differences of overall survival between MM patients with and without MPMST. **(B)** Differences of overall survival in the males between MM patients with and without MPMST. **(C)** Differences of overall survival in older patients (≥65years) between MM patients with and without MPMST. **(D)** Differences of overall survival in stage ISS III between MM patients with and without MPMST. **(E)** Differences of overall survival between MM patients and patients with MM after MPMST. **(F)** Differences of overall survival between MM patients and patients with MM before MPMST.

Furthermore, Kaplan -Meier survival analysis revealed that patients with MM and MPMST had shorter OS than MM patients without MPMST (**Figure 4A**), especially male patients with MPMST (**Figure 4B**), older patients aged ≥ 65 years (**Figure 4C**), and those with ISS stage III (**Figure 4D**). Further analysis revealed that the OS in patients with MM and secondary MPMST, but not prior MPMST, was obviously reduced compared with those without MPMST (**Figures 4E, F**). The difference in OS across different characteristics, including sex, age, and stage of ISS in patients with MM and secondary MPMSTs, was not available due to the small number of samples. These findings suggest that the male sex, advanced age (≥ 65 years) and advanced ISS stage are closely related to a poor prognosis in patients with MM and MPMST.

### Difference in the occurrence, survival and treatment of MPMSTs between patients diagnosed after MM and those diagnosed before MM

Next, we compared differences in the incidence rate, survival, and treatment in patients with MM and with MPMST diagnosed before MM (MPMST-1) and MPMSTs after MM (MPMST-2). The incidence rate of patients having MPMST diagnosed after MM were obviously lower than those having MPMST diagnosed before MM (32.35% vs. 67.65%, **Figure 1B**). In particular, patients with MPMST diagnosed after MM were more likely to be patients aged < 65 years compared with those having MPMSTs diagnosed before MM (63.6% vs. 21.7%, **Table 1**). TF2T rates of < 36 months in MPMSTs diagnosed after MM in patients with MM and MPMST diagnosed after MM were obviously higher than those having MPMSTs diagnosed before MM (72.7% vs. 34.8%, **Table 1**). The median time from the diagnosis of additional primary solid cancer after MM was 32.7 months, which was less than the median diagnosis time of MPMST before MM (63.3 months). These data suggest that MM patients might be more quickly prone to a solid cancer despite a lower incidence rate.

The OS time was similar between MM patients with MPMST occurred before MM (MPMST-1) and after MM (MPMST-2), including male patients, the older (≥65 years) and patients in ISS III stage (**Supplementary Figure S1**). Briefly, these results suggest that patients with MM and MPMST had an unfavorable prognosis compared with those without, while no significant difference in OS between MM patients with MPMSTs diagnosed after MM and before MM.

**Figure 5:**
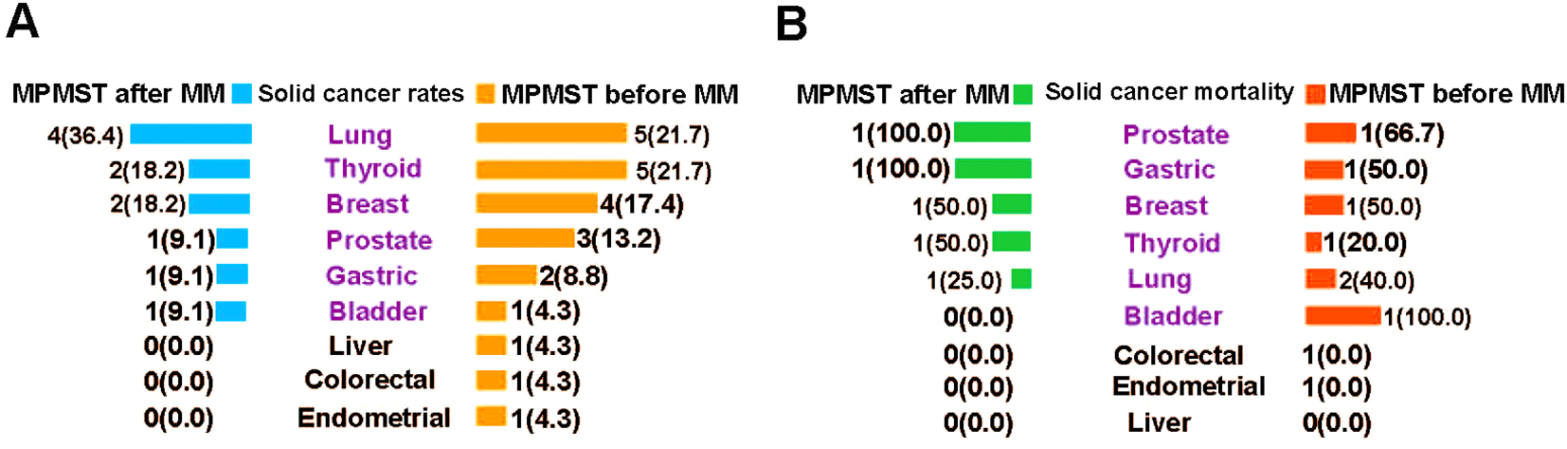
The incidence of morbidity and mortality of solid cancers in MPMSTs occurred after and before MM. **(A)** The incidence rate of solid cancers in MPMSTs occurred after and before MM. **(B)** Mortality of solid cancers in MPMSTs occurred after and before MM.

Our data indicated that the three most common solid cancers in MPMSTs diagnosed after MM and diagnosed before MM were lung cancer (36.4% vs. 21.7%), thyroid cancer (18.2% vs. 21.7%) and breast cancer (18.2% vs. 17.4%) (**Figure 5A**); these rates are similar to those of solid cancers in China (36). Other solid cancers were prostate, gastric, and bladder cancer. The mortality in patients with MM and additional solid cancer was shown in **Figure 5B**, but the number of cases for each kind of solid cancer was small, which warrants further in the multicenter studies with larger samples for validation of our findings.

Recently, secondary cancers related to lenalidomide, bortezomib, and especially CAR T-cell therapy have been reported (8–10). Our results demonstrated that there were no significant differences in MM therapy (immunomodulators, proteasome inhibitors, chemotherapy, ASCT and CAR T-cell therapy) between MM patients with or without MPMSTs (**Table 1**, **Supplementary Table S1**). Similar results were observed for patients with MPMSTs diagnosed after MM and before MM (**Supplementary Table S1**). Intriguingly, patients with MM treated with immunomodulators had a higher risk of developing an additional solid cancer (**Table 2**). However, no increased risk of the occurrence of MPMSTs in MM was found after exposure to PIs, chemotherapy, ASCT and CAR-T cell therapy (**Table 2**).

**Figure 6:**
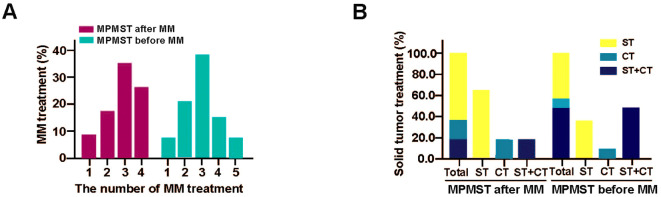
The treatment of MPMSTs occurred after and before MM. **(A)** MM treatment in MPMSTs occurred after and before MM. **(B)** Solid cancer treatment in MPMSTs occurred after and before MM. ST, surgical treatment; CT, chemotherapy.

For solid cancer treatment, surgical treatment and chemotherapy or their combination were predominant (**Figure 6**); surgery alone was the first choice for patients with MPMSTs diagnosed after MM; in contrast, surgery combined with chemotherapy was routine treatment for patients with MPMSTs before MM (**Supplementary Table S2**, **Figure 6**), demonstrating that diverse solid cancers occurred in MM and various treatment options.

## Discussion

The association and risk of secondary hematologic disease have been described among patients with MM (11–21). The effects and the risk factors of MPMST in patients with MM have not been fully investigated. This study showed that older age (≥ 65 years), higher β_2_-MG levels and the use of IMiDs instead of PIs, chemotherapy, ASCT, or CAR-T cell therapy in MM were related to an increased risk of MPMSTs. Patients with MM and MPMST including males, older adults (≥ 65 years) and patients with ISS stage III had a worse prognosis. Furthermore, the incidence rate of MPMSTs after MM is 32.35% (11/34)less than the incidence rate (67.65%, 23/34) of MPMSTs diagnosed before MM. The median occurrence time from MM to additional solid tumor was shorter than the time from primary solid tumor to additional MM. MM patients with immunomodulators rather than chemotherapy or ASCT or CAR T-cell therapy enhances the risk towards MPMSTs. Moreover, the three most common solid cancer sites in MPMSTs after and before MM were the same, including the lung, thyroid and breast.

Reportedly incidence rate of MPMSTs varies worldwide, ranging from 1.5%-12.5% (12–16, 19, 24, 25). Our results exhibited that the incidence rate of MPMSTs in MM is 1.57%, which is similar to previous data from China and Thailand but less than that identified in European and American patients, having an estimated incidence from 1.8% to 12.5% (12–14, 19, 24). This may be attributed to the discrepancies in geographical distribution, which requires a multicenter study from different countries.

Risk factors inducing two primary malignancies in a patient with MM involve host, disease and treatment-related factors. Our findings suggest that older patients (≥65 years) and higher β_2_-MG level are related to an increased risk to MPMST in MM, this is consistent with previous studies in which females and older individuals were more likely to develop MPMST (16, 25, 37), but are inconsistent with the data from Lv et al., in which the high risk cohort included males (28). Another analysis indicated that MM patients with secondary primary malignancies harbored reduced β_2_-MG levels compared with those without; whereas, their data was mixed with second hematological-related MPMSTs and had inconsistent times for collecting β_2_-MG values (16). Regularly monitoring of β_2_-MG levels is necessary for older MM patients.

A growing number of studies have revealed the treatment-related second primary hematological and nonhematological malignancies, involving lenalidomide and protease inhibitor bortezomib, particularly an association with CAR T-cell therapy (8, 10, 38). Our study revealed that patients with MM treated with IMiDs rather than PIs, chemotherapy, ASCT, or CAR T-cell therapy, are connected with an elevated risk to MPMST. Although we found an increased risk of MPMST occurring after MM treated with IMIDs because there was a discrepancy in the number of the use of IMIDs between MM and MM with MPMPST, this still require a multicenter study with large number of samples to confirm further.

IMiDs such as thalidomide, lenalidomide and pomalidomide combined with or without PIs and/or monoclonal antibodies are the most commonly used regimens for the treatment of MM (7, 39, 40). Their tumoricidal and immunomodulatory activity arise from modulation of the activity of the ubiquitin E3 ligase, Cereblon (39–41). All of these agents can bind to Cereblon and enhance the ubiquitination and degradation of IKZF1 and IKZF3, resulting in the inhibition of myeloid cell growth and derepression of IL-2 levels in T cells (39). Furthermore, Lee et al. revealed that both lenalidomide and pomalidomide have higher anti-myeloma efficacy toward dendritic cell vaccination in a MM mouse model when combined with PD-1 or PD-L1 blockade to inhibit immunosuppressive cells and restore effector cells (42, 43). Despite stable efficacy against MM, exposure to lenalidomide has been associated with an increased incidence rate of second primary malignancy, including MM and solid cancer (6, 7, 44, 45). The mechanism involved in lenalidomide-induced second primary malignancy remains complicated. Recent studies have shown that treatment with lenalidomide drives the development of secondary myeloid neoplasms and AML associated with TP53 mutation. Lenalidomide directly interacts with Cereblon, an E3 ubiquitin ligase, to ubiquitinate and degrade its substrate CK1α, facilitating TP53-mediated cell apoptosis, but thalidomide and pomalidomide do not (40, 46). It can be speculated that the TP53 mutation in patients with MM treated with thalidomide increases the risk of transformation to second primary malignancy, such as a second solid tumor. Genetic factors including TP53 mutation or gene polymorphism also increase susceptibility to second solid tumor (7, 10, 24). Furthermore, the combination of lenalidomide with melphalan may create a new tumor microenvironment that sensitizes the second primary lenalidomide-initiated malignancy (7, 46). Given the rare incidence of the TP53 mutation in our study, we did not observe a significant difference in the TP53 mutation status and effect in MM patients with or without additional solid tumor. However, genome-wide association and expression microarray analysis in patients with MM and with TP53 mutation is truly indispensable.

Intriguingly, an announcement from US Food and Drug Administration (FDA) highlights the risks caused by CAR T-cell therapy-induced T-cell cancers such as T-cell lymphoma, acute myeloid leukemia and myelodysplastic syndrome (47–51). Recent studies indicated the risk of secondary solid cancers after CAR T-cell therapy (10, 38, 52). Elsallab and colleagues identified 107 nonhematological malignancies from the data of FDA adverse event reporting system, mainly involving nervous system tumor and lung cancer, gastric cancer, skin neoplasms and breast cancer (10). Ghilardi et al. exhibited that most frequent second solid cancers after CAR T-cell therapy were skin neoplasms (nonmelanoma), non-small cell lung cancer, prostate cancer (38). Hamilton et al. detected 11 second solid cancers in 724 cases treated with CAR-T therapy, containing melanoma, prostate cancer, ductal breast cancer and lung adenocarcinoma (52). Further studies demonstrated that CAR-T therapy-related second cancers might be attributed to an integration of vector of CAR T cells into T cell’s genome causing direct tumorigenesis. Additional explanation may be associated with mutation of pivotal genes such as PBX2, JAK3, DNMT3A, and TET2, and clonal expansion (38, 53–56). We did not observe an enhanced risk to MPMST in patients with MM who had received CAR-T therapy, which can be attributed to low incidence of MPMSTs in patients with MM. It is worth noting that despite the risk in patients with MM exposed to lenalidomide and CAR-T cell therapy to develop second solid cancer, lenalidomide and CAR -T cell therapy are promising regimens for patients with MM due to their great efficiency and benefits. Patients with MM who choose CAR -T cell therapy or lenalidomide should be required to perform high -throughput sequencing and viral vector monitoring, which contributes to the identification of mutated genes and underlining risk in patients with MM.

Regarding the location of solid cancers diagnosed before and after MM, the breast, prostate, and lung were more common in American population-based surveillance (11) and the prostate, breast and colorectum were the top three sites in the Norway and Germany populations (12, 19), differing from our results that the lung, thyroid and breast as the top three sites. This discrepancy may be associated with differences in the morbidity of solid cancers in various countries. Additionally, plasmacytoma is common in MM, including solitary plasmacytoma and EMP typically found in skin and soft tissues. At relapse, common sites of plasmacytoma include liver, kidney, lymph nodes, central nervous system, breast, and pericardium, which can be misdiagnosed as the second solid tumors in patients with MM (33, 34). CT and histopathology are required to clarify the EMP and the second solid tumor and to allow precise treatment.

The limitations of our study include the small number of patients with solid primary metachronous cancers before and after MM and the lack of long-term data (15 -20 years) to support our findings. Furthermore, methods to explain impact of secondary cancers on the survival of patients with MM and MPMST should be expanded. We investigated the incidence rate of specific solid cancer in patients with MM which can be supported by previous data (12, 24), whereas the conclusions derived from the study should be carefully drawn due to the small number of samples.

In conclusion, using well-characterized individual clinical information, we found that older patients (aged ≥ 65 years) and patients treated with the immunomodulator lenalidomide rather than with protease inhibitor, chemotherapy, ASCT, or CAR-T cell therapy had a higher risk of MPMST. Patients with MM and MPMST, including male patients, older patients (≥ 65 years) and advanced patients with ISS stage III, had a higher survival risk, although we did not observe significant differences between MPMSTs occurring before or after the diagnosis of MM.

## Data Availability

The raw data supporting the conclusions of this article will be made available by the authors, without undue reservation.
