# Supplementary material for: Effect of metachronous primary and secondary solid cancers in patients with multiple myeloma: a retrospective study from a single-center
Source: Front Immunol. 2025 Mar 10;16:1516471. doi: 10.3389/fimmu.2025.1516471 (PMC11931010; doi:10.3389/fimmu.2025.1516471)
Supplement: Supplementary file 1 [file DataSheet1.docx]

**Supplementary Figure and Tables for**

**Effect of metachronous primary and secondary solid cancers in patients with** **multiple myeloma: a retrospective study from a single-center**

Yunfei Ji^1, 2, 3#^, Hujun Li^1, 2, 3#^, Huanxin Zhang^1, 2, 3^, Hai Cheng^1, 2, 3^, Ying Wang^1, 2, 3^, Kailin Xu^1, 2, 3^, Zhenyu Li^1, 2, 3^

1Blood Diseases Institute, Xuzhou Medical University, Xuzhou, Jiangsu, China

2Department of Hematology, The Afﬁliated Hospital of Xuzhou Medical University, Xuzhou, Jiangsu, China

3Key Laboratory of Bone Marrow Stem Cell, Xuzhou, Jiangsu, China


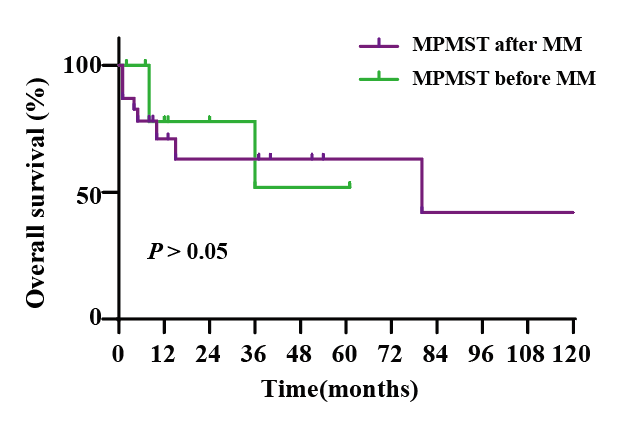


**Supplementary Figure 1.** Kaplan-Meier analysis of overall survival of MPMSTs occurred after and before MM.

**Supplementary Table 1** MM treatment in MPMSTs diagnosed after and before MM

| **Treatment** | **MPMST after MM (n =11, %)** | **MPMST before MM (n =23, %)** |
| --- | --- | --- |
| Proteasome inhibitor | 10(90.9) | 17(73.9) |
| Immunomodulator | 7(63.6) | 17(73.9) |
| Chemotherapy | 9(81.8) | 17(73.9) |
| ASCT | 3(27.3) | 5(21.7) |
| CAR-T cell therapy | 2(18.2) | 4(17.4) |
| numbers of treatment |  |  |
| 1 | 1(9.1) | 2(8.6) |
| 2 | 2(18.2) | 5(21.7) |
| 3 | 4(36.3) | 9(39.1) |
| 4 | 3(27.3) | 4(17.3) |
| 5 | 0(0.0) | 2(8.7) |
| untreated | 1(9.1) | 1(4.3) |
| Note: ASCT: autologous stem cell transplantation; CAR-T cell therapy: Chimeric antigen receptor T cell therapy. | | |

**Supplementary Table 2** Solid cancers treatment in MPMSTs diagnosed after and before MM

| **Treatment** | **MPMST after MM (n =11, %)** | **MPMST before MM (n =23, %)** |
| --- | --- | --- |
| ST | 5(45.4) | 9(39.1) |
| CT | 2(18.2) | 2(8.7) |
| ST+CT | 2(18.2) | 11(47.8) |
| untreated | 2(18.2) | 1(4.4) |
| Note: CT: chemotherapy; ST: surgical treatment. | | |
